# Male students’ experiences on predictors of waterpipe smoking reduction: A qualitative study in Iran

**DOI:** 10.18332/tpc/112249

**Published:** 2019-09-30

**Authors:** Saeed Bashirian, Majid Barati, Fazlolah Ahmadi, Hamid Abasi, Manoj Sharma

**Affiliations:** 1 Social Determinants of Health Research Center, Hamadan University of Medical Sciences, Hamadan, Iran; 2 Nursing Department, Faculty of Medical Sciences, Tarbiat Modares University, Tehran, Iran; 3 Department of Public Health, School of Health, Hamadan University of Medical Sciences, Hamadan, Iran; 4 Behavioral & Environmental Health, School of Public Health, Jackson State University, Jackson, United States

**Keywords:** students, multi-theory model, waterpipe smoking, directed content analysis, Iran

## Abstract

**INTRODUCTION:**

In recent years, waterpipe smoking (WPS) has increased among adolescents in Iran. This study aimed to explain the experiences of high school students in Iran on predictors of WPS reduction based on a multi-theory model (MTM) of health behaviour change.

**METHODS:**

This study was a qualitative study of directed content analysis that was conducted in high school male students in Hamadan, Iran, in 2017. In this study, 34 students who had smoked waterpipe (WP) in the last month were recruited through snowball sampling that was continued until data saturation. The data were collected through semi-structured, individual interviews and were then analyzed using directed qualitative content analysis.

**RESULTS:**

The data analysis resulted in the extraction of 104 final codes around the six themes of predetermined MTM constructs consisting of participatory dialogue, behavioural confidence, changes in the physical environment, emotional transformation, practice for change, and changes in the social environment. The findings of this study showed that this model has the potential to explain the behaviour of WPS reduction. The main predictors of reduction in WPS are behavioural confidence, social environment change, and participatory dialogue.

**CONCLUSIONS:**

Findings of the research showed that the belief in an individual’s ability, support from friends and the benefits of WPS reduction are the most important factors in reducing WPS among students. Therefore, it is suggested that comprehensive interventions be developed to improve the individual and social factors that are effective in WPS reduction.

## INTRODUCTION

Waterpipe smoking (WPS) is the practice of inhaling tobacco smoke generated by a multi-stemmed device. Generally, charcoal pieces are placed on top of a perforated aluminium foil separating it from specially made tobacco that is usually flavored^1^. This entails the use of tobacco in centuries-old tradition through what is differently named as hubble-bubble, waterpipe, hookah, or narghile^2^. WPS has multiple harmful effects. For example, lung cancer, respiratory diseases, low birth weight, periodontal disease, bladder cancer, nasopharyngeal cancer, oesophagal cancer, oral dysplasia, infertility and hepatitis C infection^3^ are all attributed to WPS. It is estimated that approximately 100 million people, particularly teenagers, use this kind of smoking^4^. Since the period 2009–2016, studies reporting on WPS indicated a 0.4% to 2.9% annual increase in current WPS in the Eastern Mediterranean and European Regions, and 0.3% to 1.0% increase in the USA^5-7^. Population data indicate that from 3.3% to 7.5% of US youths have tried waterpipe^8^.

Studies reported a high prevalence of WPS use among adolescents globally^9,10^. In the Mediterranean, the use of WPS is an important public health problem. There is a potential for increased usage and hence increased tobacco-related morbidity and mortality unless intervention measures are taken now^11^. In a study, 9.7% of the students consumed waterpipe in the past month, of which 66.6% were male and 33.4% were female, which indicates a higher prevalence in males^4^. In another study, the prevalence of current WPS was reported as 26.3% among male high school students^12^.

In general, factors such as the pleasant smell, the attractive design of the waterpipe, availability and affordability, social acceptance due to cultural views and beliefs that WPS is less harmful compared to cigarette smoking influence WPS among adolescents^4^. Students who smoke waterpipe have low self-efficacy to quit and find it cost-effective. Other factors associated with its use include enjoyment with friends, loss of friends if one was to quit, feeling unwell if one was to quit, and feeling of loneliness on quitting. Also, students believe that WPS is related to some factors such as high perceived rewards like a sign of manhood, sense of pleasure, filling the vacuum of loneliness, focusing more on doing things, increasing intellectual capability, becoming calm, getting friends together, and accepting friends^13^. Evidence suggests that there are different structural and social factors in shaping behaviour patterns of cigarette smoking and WPS, which requires behavioural change theories to analyze these behaviours. To achieve efficacy and effectiveness in WPS reduction, educational programs are needed for developing healthy behaviours^14,15^.

Although a variety of theoretical models have been used to identify such factors, the existing health behaviour theories and models have conceptual problems, lack predictive power, are not parsimonious, and/or are too comprehensive, and consequently, impractical. In recognition of these issues, Sharma^16^ recently proposed a multi-theory model (MTM) for health behaviour change, using constructs that have been extensively validated with a broad range of populations in cross-cultural settings. The MTM poses that three primary constructs explain and predict the initiation of health behaviour change including participatory dialogue (the advantages being more than disadvantages of a health behaviour change), behavioural con-fidence (related to perceived behavioural control and self-efficacy) and changes in the physical environment (obtainability, availability, accessibility of resources). Also, three additional constructs in sustenance or maintenance of behaviour including emotional transformation (controlling and directing feelings toward goals), practice for change (supervision and reflection on behaviour) and changes in the social environment (the role of friends and family in dealing with change)^16-18^. Given that WPS is complex and rooted in people’s beliefs, the use of qualitative methods can lead to in-depth information from waterpipe consumers^19^. Therefore, this study aimed to explain the experiences of high school students in Iran on predictors of WPS reduction based on MTM.

## METHODS

### Participants

In this study, the first- and second-grade high school male students of Hamadan city in Iran, who had experienced WPS in the last month, were included. At first, one school was selected from the upper part of the city and one from the lower city of Hamadan. After interviewing the first student who had a history of WPS in the past month (purposive sampling), he was asked to introduce his other friends who had smoked WP in the past month to the researchers (snowball sampling). Selection of male students to obtain different views and perceptions was sought with the highest diversity (in terms of age, academic background and high school position in different areas of Hamadan). In this study, data were saturated after 34 interviews.

### Procedure

The present study was qualitative and conducted using directed content analysis. To collect data, individual interviews were used during a 3-month period March–July 2017. The interviews were conducted in one of the classes by an interviewer and a note-taker. All of the interviews and translations were conducted in Persian. In this type of qualitative research, since there are no established criteria for determining the number of contributors to the qualitative research before the study begins, this number is based on the information obtained and until the total saturation of the classes has occurred (when other contributors have new information to add to the data already collected, and there is repetition of the themes)^20^. To observe ethics in the research, researchers explained the reason for recording male students’ voice during the interview and emphasized that all information received was confidential and was to be used solely for the purpose of the study only. During the interviews, a gift (notebook) was provided as an incentive. Also, researchers emphasized that the participants had the right to withdraw from the study at any time. Written consent was received from the people interested in participating in the study. The present study was approved by the Hamadan University of Medical Sciences Ethics Committee (ID: IR.UMSHA. REC.1396.21).

### Instrument

The interviews were conducted using the MTM constructs, which included participatory dialogue, behavioural confidence, changes in the physical environment, emotional transformation, practice for change, and changes in the social environment. All of the interviews lasted 20–40 minutes. Questions were open-ended such as: ‘What do you think of the disadvantages and advantages of WPS reduction?’, ‘How sure are you that you can reduce WPS?’, ‘How sure are you that you can avoid WPS environments?’, ‘How sure are you that you can direct your feelings about WPS reduction?’, ‘How sure are you that you can supervise your WPS by keeping a diary?’, ‘How sure are you that you can get help from friends and family for WPS reduction?’. Also, in the interviews some in-depth probing was conducted such as: ‘Would you explain more?’, What do you mean?’, ‘Can you give an example?’. (Table 1).

**Table 1:** Guidelines for the interviews with the participants

*The MTM constructs*	*Questions*
**Participatory dialogue**	What do you think about the disadvantages and the benefits of WPS reduction?
**Behavioural confidence**	How sure are you that you can reduce WPS?
**Changes in the physical environment**	How sure are you that you can avoid WPS environments?
**Emotional transformation**	How sure are you that you can direct your feelings about WPS reduction?
**Action for change**	How sure are you that you can supervise your WPS by keeping a diary?
**Changes in the social environment**	How sure are you that you can get help from friends and family for WPS reduction?

### Data analysis

One of the methods of qualitative research, which is presented in 2005 by the Hsieh and Shannon analysis, is a directed content analysis method or theory-based content analysis method^21^. In the directed content analysis method, initial coding begins with an established theory or results. This type of analysis aims to validate the theory or develop a conceptual framework. The theory chosen in this type of study can help in focusing the research question. On the other hand, theory can help in predicting interesting variables or relationships between variables. As a result, it is also useful in determining how the initial encodings occur and the relationships between the codes^22^.

After recording a participant’s voice, the text of the interviews was written on paper at the first opportunity by two researchers (in the field of health education and promotion). Given that in qualitative research, researchers must be immersed in the information^21^, the interviews were heard several times and their transcripts were repeatedly reviewed. In this research, different methods were used to provide accreditation and analysis of data such as prolonged engagement in the information gathered and reading manuscripts multiple times and member checking method for comparing the researchers’ and views of the participants. After several accurate readings, the text was analyzed by the researcher as an open coding system for the production of primary category. For this purpose, the texts of the interviews were first divided into semantic units and then summarized and converted into codes. Different codes were compared based on their similarities and differences and divided into categories. At this stage, the first classes were discussed and reviewed by three researchers to reach the themes^23,24^.

In this study, we identified and categorized the whole content as it related to the particular phenomenon of our study. The entire text was studied, and those sections that were identified through the initial recognition by the researchers were noted. In the next step, based on predetermined codes (based on theory), the parts were marked and encoded.

To provide and validate data credibility, there was a constant relationship with the male students to gain a better perception of their opinions. To confirm the coding method in the MTM constructs, a review was carried out by supervisors (three experts in the field of health education and promotion). The supervisors did not consider their own personal views about WPS (say, anti-smoking) in their research.

## RESULTS

In this study, 34 male students in grades 8–12 with a mean age of 16.47 years were studied. The mean age of initiating WPS was 11.6 (2.83) years (Table 2). After identifying the basic concepts, the initial codes were extracted from the interviews. These codes were classified and sorted according to their compatibility and similarity and were placed in the primary and secondary classes of the main structures of the MTM. These structures or themes based on the MTM included: participatory dialogue, behavioural confidence, changes in the physical environment, emotional transformation, practice for change, and the changes in the social environment. Out of 34 interviews, 621 primary codes were extracted and carefully evaluated. Then 104 final codes were extracted, which were reduced to 41 codes. In the next stage, by studying the main codes, 13 subcategories and 7 main categories were extracted (Table 3).

**Table 2:** Demographic characteristics of the study participants (n=34)

*Variables*	*Range*	*n (%)*	*Mean±SD*
**Age (years)**	14–16	12 (35.3)	
	16–18	22 (64.7)	
**School grade**	8–10	20 (58.8)	
	10–12	14 (41.2)	
**Age of initiating WPS**	6–9	3 (8.8)	11.6±2.83
	9–12	15 (44.2)	
	12–15	10 (29.4)	
	15–18	6 (17.6)	
**Weeks since last WPS**	1	10 (29.4)	
	2	9 (26.5)	
	3	10 (29.4)	
	4	5 (14.7)	

**Table 3:** Display of the process of extracting a theme from semantic units, codes, and related categories

*Theme*	*Category*	*Subcategory*	*Code*	*Semantic units*
Participatory dialogue	*Perceived advantages*	Be healthier	Fewer physical complications	You get better, you do not have a headache, and your blood pressure is set, your whole body gets less damaged, your illness falls.
		Lower cost	Save money in your pocket	You have low cost, do not waste money, you have savings.
			Saving time	If you save time, you have more time, do not waste time.
			Have a healthier friend	You meet friends, whom you look for, do not like a friend, you find a new friend.
		Better social image	Family acceptance	You are proud of your predominance. You do not blame your family. Your family will be better off.
			Doing better homework	Trying to learn more about homework.
	***Perceived disadvantages***	Lower fun	Lose friend	If I let him down, I do not have to be with my comrades, my friends are getting away.
			Losing fun	Have fun with all the Iranian youths. Other fun for us. This is a great place to relax. It’s fun.
		Physical and psychological dependence	The physical dependence on nicotine	I’m annoying. I’m so angry with my nicotine. Well, you’re addicted to being bullied like a headache.
			Psychological dependence on nicotine	The disadvantage of this is when you will only be living and doing it.
			Believing in individual ability	If I do not, I will not cut it if I do not want to.
**Behavioural confidence**	*Self-efficacy*	Strong will and belief	Resist temptation	If I go in front of my friends and even the conditions and availability of WP is possible, I can reduce it or less to beat it.
**Physical environment change**	*Context suitable for consumption*	Availability	More supply	Get some WP anywhere and stay tuned.
		Cheapness	Low price	WP is not expensive. With a little money, he can hookah.
**Emotional transformation**	*Pleasant feeling WPS*	Enjoyment of consumption	Better mood due to consumption	Make me feel so trickier to make me feel better.
		Enjoy preparing	Excitement for making WP	Making and preparing for it has a special pleasure.
**Action for change**	*Active reflection and reflective behaviour*	Monitor the WPS	Having an incentive due to the amount of consumption	I wrote this to the energy editor. I was happy to have this writing a jolt.
**Social environment change**	*Social support*	Emotional support	Compassionate friend	My relationship with my friends is high. I have a relationship with them so that I can use their knowledge and understanding to reduce my WPS.
		Informational support	Awareness of the friend’s side	I have a friend who says that this is the case. Any advice or advice he makes is important.

### Participatory dialogue about WPS reduction

The participatory dialogue structure has already been divided into two main categories of perceived advantages and disadvantages of WPS reduction. Most of the students believed that WPS reduction has benefits such as being healthier, lower costs and having a better social image:

*‘The benefits of WPS reduction are to breath better, you do not have a headache or hypertension’.* (Participant 4, P4, aged 17 years, 11th grade, electronics field)

*‘I knew someone that wanted to smoke waterpipe, but did not smoke at that time to save his money. Two years later he saved a lot of money, also saving time is a benefit of WPS reduction.’* (P6, aged 17 years, 11th grade, electronics field)

*‘If you reduce WPS, you have a better image in your family. Everything you want, they provide for you due to reducing WPS and reducing WPS causes reducing bad friends.’* (P3, aged 16 years, 10th grade, utility field)

*‘Reducing WPS helps you to do school homework better and you have good scores in the exams.’* (P21, aged 14 years, 8th grade)

They also believe that less fun and physical and psychological dependence are the disadvantages of WPS reduction:

‘If I reduce WPS, I will not be able to hang out with my friends. My friends split off, and I cannot see them.’ (P12, aged 17 years, 12th grade, electronics field)

*‘Iranian adolescents’ fun is just WPS. In our city adolescents have one fun, and it is WPS, so reducing WPS means to loss our fun.’* (P8, aged 18 years, 11th grade, automobile mechanics field)

Students believed that reducing WPS causes one to suffer from physical and psychological dependence:

*‘In fact, the disadvantages of reducing WPS are to suffer from dependence signs, for example having physical pain.’* (P19, aged 17 years, engineering field)

*‘If I reduce WPS, I will suffer from mental dependence signs, for example, intensive temptations.’* (P16, aged 17 years, automobile mechanics field)

### Behavioural confidence about WP reduction

The self-efficacy is the main category of the behavioural confidence theme. The most important and influential factor in predicting the decrease in WPS in terms of students was the will and belief in individual ability to reduce and stop WPS. They believe that the individual’s ability and will in the addiction issue, especially the WPS, play a more critical role than other factors, and one can begin to reduce the consumption of waterpipe from the will and beliefs of the individual:

*‘In my opinion, family and friends cannot help us to reduce WPS. Absolutely, the reduction of WPS is dependent on our ability and willingness.’* (P15, aged 18 years, 11th grade, automobile mechanics field)

*‘I can reduce and quit WPS. Generally, whenever I want to reduce WPS, I can, and it is not difficult for me to reduce it.’* (P34, aged 17 years, 11th grade, electronics field)

### Changes in the physical environment about WPS

WPS is inexpensive and able to afford it were the main categories of changes in the physical environment theme. Students believed that affordability, availability, and ability to buy waterpipe over other tobacco products such as cigarettes was a boost at the start and played a preventive role in reducing consumption:

*‘I think WPS is better than other options, for examples cigarette smoking and tobacco due to availability and easy to reach.’* (P2, aged 17 years, electronics field)

Students believed that waterpipe being inexpensive increases WPS in society:

*‘WPS is not expensive, and you can buy it at a low price. I think WPS cheapness causes that every adolescent starts to smoke it. In my opinion, cheapness does not help you to reduce it.’* (P25, aged 16 years, 9th grade)

### Emotional transformation about WPS reduction

The pleasant feeling is the main category of the emotional transformation theme. Most students believed that the use of waterpipe would create a positive and supportive mood:

*‘I smoke waterpipe, so that I feel better. When I smoke waterpipe, I relax and enjoy it.’* (P22, aged 16 years, 9th grade)

*‘Actually, preparing and providing waterpipe is enjoyable for me and it gives me a good feeling. I like that mood.’* (P31, aged 18 years, 11th grade, automobile mechanics field)

### Practice for change about WPS reduction

The active reflection and reflective behaviour is the main category of practice for change theme. Students believed that using notes and memorizing, was considered an effective monitoring method to help reduce WPS:

*‘Writing the amount of WPS, using notes and memorizing can help you to reduce WPS. It helps you not to forget the amount of WPS. I think it is best idea for reducing WPS.’* (P7, aged 17 years, 11th grade, electrical engineering field)

*‘In my opinion, keeping a diary is useful for reducing WPS. It makes you feel better and gives you motivation for reducing. Also, the memorizing about the amount of smoking has its good points.’* (P33, aged 18 years, 11th, automotive mechanics field)

### Changes in the social environment about WPS reduction

The social support is the main category of changes in the social environment theme. Most of the students believed that the role of friends and supporters, who could assist in terms of information and guidance on the side effects of waterpipe, play a vital role in reducing WPS:

*‘I think the most important thing about friends is their kindness, irrespective of being a smoker or not. A friend can give some advice about several harmful effects of WPS. His advice is so important for me, as in giving knowledge to me about WPS risks.’* (P26, aged 16 years, 9th grade, automobile mechanic field)

Students believed that as much as a friend could play a role in initiating and encouraging consumption, he could play a supportive role in reducing the issue and leaving the WPS:

*‘In fact, some of my friends can help me in reducing my WPS. I can rely on them due to their kindness and attention. I get on well with them and have a good relationship. I am not worried about reducing WPS, because I have helping friends.’* (P3, aged 17 years, elementary school student, electronics field)

*‘I think the best friend who can help to reduce waterpipe is if he is not waterpipe smoker. If my friends do not smoke waterpipe, I can get help for my reducing waterpipe.*’ (P18, aged 18 years, elementary school student, automobile mechanic field)

## DISCUSSION

The findings of the present study showed that WPS reduction could be explained using MTM. According to the results of this study, most of the students understood the benefits of WPS reduction and stated that reducing the WPS has advantages such as being healthier, lower costs and having a better social image.

Smokers’ beliefs about the benefits of quitting are related to smoking cessation behaviour in smokers motivated to quit smoking^25^. Concerning this finding, the results of the same study showed that emphasis on benefits of reducing WPS behaviour (being healthier, lower costs) could have an active role and help to reduce WPS^26^. Therefore, educational interventions should emphasize the positive outcomes and benefits of adopting health behaviour or correcting negative health behaviour.

In connection with the disadvantages of reducing WPS, students said that if they reduce WPS, their entertainment will be lowered, they would lose their friends, and their leisure will be less. They also reported suffering from complications of quitting WPS that can be physical and mental.

In general, those who use tobacco emphasize the perceived disadvantages can be a motivational predictor of the quitting and related factors^27^. The results of other studies have shown that, besides being friends, keeping in touch with friends, being afraid of getting lonely and attracting friends are reasons why waterpipe smokers are turning to it and will not leave it^13,28^. In our study, suffering from complications of quitting (physical and mental) was a new result that other studies have not mentioned, so it seems WPS is not only a tobacco smoking behaviour but also a kind of social behaviour that waterpipe consumers use to get together with friends and have enjoyable moments. Therefore, WPS is suggested and presented within a community of fun and lively entertainment aimed at getting together for young people to spend pleasurable moments. The results of our study showed that students believed that the will and belief in individual ability (behavioural confidence or self-efficacy) played an essential role in reducing and leaving the waterpipe.

Self-efficacy is an estimate of the degree to which a person has the confidence to perform a particular behaviour or a chain of specific behaviours to control and manage situations^29^. Along with our result, the results of various studies have shown that self-efficacy and belief in individual ability play an important role in the behaviour of WPS^26,28,30^. It seems that by determining the level of self-efficacy of waterpipe smokers, self-efficacy enhancement strategies can be of great importance through the motivation and continuity of the behaviour, saying-no skill, the increase in self-esteem, and self-belief in one’s ability to reduce and quit the behaviour.

Other results from this study were that students believed lower expense, availability, and ability to buy waterpipe had a significant role in WPS in the community. The physical environment in the discussion of drug use and tobacco behaviour can include the ability to access, accessibility, and provision of resources easily^17^. Along with our results, recent studies have shown that the role of facilitating factors such as availability, price and ability to buy, increase the prevalence of WPS in the community^19,31-33^. It can be deduced that comprehensive policies and measures from health policymakers need to be taken to prevent, reduce or quit WPS, enhance or modify the physical environment including the removal of all signs and stimuli from WPS.

The sense of relaxation of the preparation and the pleasure of WPS was one of the other results that were reported by students. Emotional changes, the ability to direct emotions and guide them towards the goal is important for helping to change health behaviour^33^. Various studies consistent with the results of our study have shown that the sensory properties of WPS include taste and smell^33-35^. Our study showed that the sense of relaxation in the preparation of the waterpipe creates a good mood that promotes smoking waterpipe. The tendency and feeling of people regarding WPS can be rooted in their desired attitude. Therefore, it seems that in educational interventions, based on the health risks of WPS, a positive attitude can change to a negative attitude towards WPS.

Another finding of this study was that students considered effective monitoring methods, to help reduce WPS, memos and mentoring. They argue that their monitoring of the behaviour of their WPS can help reduce WPS. Self-regulation means a tendency to control internal modes, control momentums, and behaviours, and adapt them to criteria to achieve the goal^36^. Consistent with the results of our study, the study by Evans et al.^37^ showed that students who had less self-regulated feelings than other students had a higher chance of starting smoking. Regarding the lack of studies in the field of the role of self-regulation in WPS, it seems that using the self-regulating process in students can create changes in them and direct them in reaching their goal, so that the probability increases for their behaviour to change to reduce waterpipe.

This study showed that the role of a friend in emotional support that is associated with compassion and helps reduce WPS, is one of the principles of social support and this can be important in reducing WPS and plays a significant role. Social support is the degree to which people give it to close relatives or to those in need in critical situations; this includes information, emotional support, material support, and feedback support^22^. Consistent with the results of our study, the study by Kong et al.^38^ showed that the use of social support could reduce the chance of smoking throughout the life of adolescents. Also, another study found that the role of friends and family members in WPS and cigarette smoking is important, and they can play an influential role for consumption when starting and continuing WPS^39^. Therefore, in educational planning and interventions, it is necessary to pay attention to the friends of the waterpipe smoker and to inform them about the harmful effects of WPS so that they also inform their friends about the harm caused by WPS.

### Limitations

This study has some limitations. First, this study is qualitative and limited to grade 8–12 high school students in Hamadan. Being qualitative it provides a snapshot in time and does not generalize to all community adolescents. Second, our study was not implemented among female students, because of challenges of getting permission to enter girls’ high schools. Thus, implementation of the program to cover female students could give better estimates of WP use and associated factors among all Iranian adolescents. Third, the present study’s design is limited in being able to draw a definitive conclusion regarding efficacy.

## CONCLUSIONS

The findings of this study indicate that WPS by students based on MTM is more influenced by the behavioural confidence, social environment and participatory dialogue constructs using a qualitative approach. In general, behavioural confidence, the role of student friends and awareness of the benefits of reducing WPS play an important role in adopting behaviours related to WPS. Therefore, health planners must design and implement comprehensive interventions in schools in order to improve individual and environmental factors in order to reduce WPS.
